# REGEN: Ancestral Genome Reconstruction for Bacteria

**DOI:** 10.3390/genes3030423

**Published:** 2012-07-18

**Authors:** Kuan Yang, Lenwood S. Heath, João C. Setubal

**Affiliations:** 1 Virginia Bioinformatics Institute, Virginia Polytechnic Institute and State University, Blacksburg, VA 24061, USA; E-Mail: yangkuan81@gmail.com; 2 Computer Science Department, Virginia Polytechnic Institute and State University, Blacksburg, VA 24061, USA; E-Mail: heath@vt.edu; 3 Department of Biochemistry, University of São Paulo, São Paulo 05508-000, Brazil

**Keywords:** bacterial genome reconstruction, phylogeny, neighboring gene pairs, evolutionary bioinformatics

## Abstract

Ancestral genome reconstruction can be understood as a phylogenetic study with more details than a traditional phylogenetic tree reconstruction. We present a new computational system called REGEN for ancestral bacterial genome reconstruction at both the gene and replicon levels. REGEN reconstructs gene content, contiguous gene runs, and replicon structure for each ancestral genome. Along each branch of the phylogenetic tree, REGEN infers evolutionary events, including gene creation and deletion and replicon fission and fusion. The reconstruction can be performed by either a maximum parsimony or a maximum likelihood method. Gene content reconstruction is based on the concept of neighboring gene pairs. REGEN was designed to be used with any set of genomes that are sufficiently related, which will usually be the case for bacteria within the same taxonomic order. We evaluated REGEN using simulated genomes and genomes in the Rhizobiales order.

## 1. Introduction

Ancestral genome reconstruction can be understood as a phylogenetic study of a set of species with more details than what is provided by a traditional phylogenetic tree. It may include information about ancestor species such as their gene content, the order of these genes in the genome, the replicon architecture, and the nucleotide sequence itself. Such information, when reliable, can help us better understand the evolutionary history of a set of organisms and thereby shed light on the genomic basis of phenotypes. Ancestral genome reconstruction in this sense and for prokaryotic genomes is the topic of this work.

Boussau *et al*. [1] reconstructed ancestral gene sets for a number of *α*-proteobacteria and quantified the flux of genes along the branches of the species tree. They inferred that the last common ancestor (LCA) of the *α*-proteobacteria was a free-living, aerobic, and motile bacterium with pili and surface proteins for host cell and environmental interactions. In that work however the authors did not attempt any reconstruction beyond gene content and overall genome size based on number of genes. Slater *et al*. [2] proposed genome reconstructions for organisms in the Rhizobiales, Vibrionales, and Burkholderiales orders. Their scenario for the Rhizobiales, based on extensive comparative genomic analysis, posits an LCA genome with one chromosome and one plasmid. From this ancestor, several paths followed, some in the direction of enlarging the ancestral plasmid until it became a chromosome (*Agrobacterium tumefaciens* C58 and *Agrobacterium vitis* S4), and some in the direction of incorporating the plasmid into the chromosome (*Mesorhizobium loti, Bradyrhizobium japonicum*), with other intermediary cases. However, this reconstruction focused on small sets of genes and was essentially performed manually.

Automated methods for ancestral genome reconstruction fall into two main categories, phylogeny-based methods and genome rearrangement-based methods. Two of the best known phylogeny-based methods are the Sankoff algorithm [3] and the Fitch algorithm [4]. Although both algorithms are designed for ancestral nucleotide sequence inference, they can be adapted for gene order inference with a few modifications. For example, an ancestral gene reconstruction method based on neighboring gene pairs (NGPs) has been proposed [5]. An NGP is a pair of genes physically adjacent to each other on a replicon. The technique proposed by Bhutkar *et al*. [5] extracts NGPs from genomes of extant species. The method then determines the occurrence of these NGPs in the ancestral genomes and outputs a list of conserved blocks assembled from the NGP content for each ancestor. The fundamental assumption of the method is that if adjacent homologous genetic loci are observed in both child species, then it is highly likely that they are also adjacent in the parent species. NGP-based methods can reconstruct ancestral genomes with thousands of genetic loci and have no limitation on allowed evolutionary events [5].

Compared to phylogeny-based methods, genome-rearrangement-based methods usually start by simplifying genomes into strings of symbols, each of which representing a gene. Homologous genes are represented with the same symbol. No duplications are allowed in most of these methods and different heuristics are used to ensure this restriction. This group of methods is computationally demanding, as reconstructing a phylogeny from gene order data is NP-hard [6,7,8]. Although various heuristic methods have been developed [9], they are only applicable to small and medium-sized data sets [5]. Furthermore, it has been suggested that this category of methods needs further study before they can yield reliable results in ancestral genome reconstruction [10,11].

The rapid accumulation of numerous sequenced genomes requires detailed ancestral genome reconstruction methods that not only take into account several kinds of information available in annotated genomes but also are scalable to large numbers of genomes. Here we present a computational system that we call REGEN (REconstruction of GENomes), which includes phylogenomic tree reconstruction, ortholog identification and refinement (which uses results previously published [12]), gene content reconstruction, contiguous gene run reconstruction, and replicon architecture reconstruction. It does not include nucleotide sequence reconstruction.

Our work is the first attempt to perform NGP-based ancestral genome reconstruction at the gene, replicon, and whole genome levels in a fully automated fashion permitting both maximum parsimony (MP) and maximum likelihood (ML) criteria. With the NGP-based method at its foundation, REGEN is capable of performing ancestral genome reconstruction on dozens of genomes with thousands of features. The ability to infer the replicon configuration of ancestral genomes automatically without prior assumptions is also an innovation.

The performance of REGEN was evaluated by running it on simulated datasets using a simulator that we developed [13] and by running it on real genomes and comparing its results with previous studies. We applied REGEN to a group of Rhizobiales species that vary significantly in life style (e.g., plant pathogens, animal pathogens, mutualists, and free-living bacteria), genome architecture (e.g., single chromosome, pair of chromosomes, with and without plasmids, and large and small plasmids), and genome size. It is important to point out that REGEN was designed to be used on any group of genomes that are sufficiently related, which will usually be the case for bacteria within the same taxonomic order.

**Figure 1:**
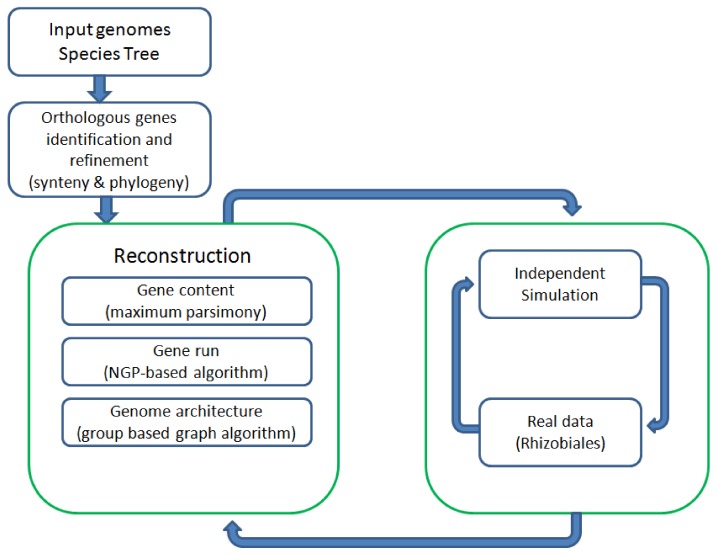
Overview of all major components in REGEN.

Figure 1 shows all the major components of REGEN and their relationship to each other. The system takes as input a collection of annotated genomes and a species tree for these genomes. REGEN is open source software [14].

## 2. Results and Discussion

### 2.1. Results on Simulated Data

We used our own simulator (see Section 3.9 to evaluate REGEN and choose values for certain reconstruction parameters. In the simulation run we defined the starting genome (the LCA) with a main chromosome of 1,000 genes and a plasmid of 200 genes. Twenty separate simulations were carried out. In each simulation the resulting phylogeny resulted in 21 final genomes of which two were considered out-groups.

We compared reconstructions produced by the MP and ML methods with gene pair cutoff set to 0.75, 0.8, 0.85, 0.9, 0.95, and 0.97. Gene occurrence cutoff was set to 0.9 in all ML reconstructions. All the numbers shown in the tables that follow are averages calculated from all of the reconstructions with the same parameter set over the 20 simulated data sets. Evaluation with simulated data includes genome coverage, longest reconstructed gene run length, conserved block reconstruction, gene pair precision versus recall measure, and replicon reconstruction accuracy. Based on all the benchmarks we obtained using simulated data, we set the gene pair cutoff to 0.9 for using REGEN on real genomes. Details can be found in the supplementary material.

### 2.2. Evaluation with Operon Structure Information

To evaluate REGEN’s performance on real genomes, we used operon structure information [15] to validate the reconstructed gene runs for ancestral genomes. Operon information is also in the form of gene pairs, which will be referred to as operon gene pairs from now on.

**Figure 2:**
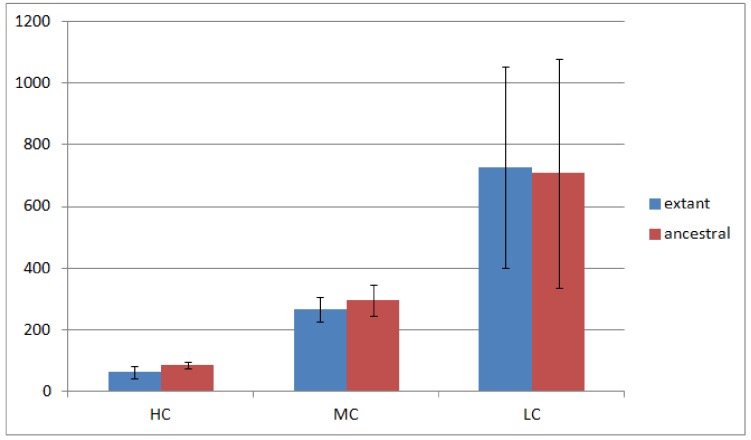
Number of operon gene pairs in extant and ancestral genomes. The error bars represent standard deviation.

Of the 23 Rhizobiales genomes we have analyzed, 19 have operon gene pair predictions. We divided all operon gene pairs into three groups by the number of genomes they occur in. The highly conserved (HC) group contains 184 operon gene pairs occurring in 10 or more genomes; the moderately conserved (MC) group contains 688 operon gene pairs occurring in 6 to 9 genomes; and the less conserved (LC) group contains 3,792 operon gene pairs occurring in 2 to 5 genomes. Operon gene pairs occurring in only a single genome are not considered due to lack of conservation. The overview of the occurrence of all operon gene pairs is shown in Figure 2.

Figure 2 shows that the number of operon gene pairs in classes MC and LC are similar in ancestral and extant genomes. However the number in HC is significantly higher (two tailed t-test, *p*-value 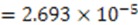) in ancestral genomes as compared to extant genomes. The number of HC operon gene pairs is also correlated (*r* = 0.81, correlation test) with the level of the ancestor, which is defined as distance in number of edges that the ancestor species has from the extant species. We believe this result is due to the fact that highly conserved gene pairs in input genomes are more likely to be reconstructed as present in ancestral genomes. We also examined the status of the reconstructed gene pairs and gene runs in the reconstructed LCA of Rhizobiales in terms of operon gene pair support. We say that a gene run is *supported* if 60% or more of all the gene pairs involved are operon gene pairs. By using this criterion, 228 out of the total 305 reconstructed gene runs in the LCA genome are supported. Furthermore, out of the total 1016 reconstructed neighboring gene pairs in the LCA genome, 770 are operon gene pairs, which also supports our expectation.

### 2.3. Leave-One-Out Test

We carried out a series of leave-one-out tests to examine the stability of our reconstruction method. We performed 22 different ancestral reconstructions of the Rhizobiales data set at gene pair cutoff = 0.85 and 0.9 with each one of the Rhizobiales genomes left out. In order to simplify the analysis process, we focused on the reconstructed gene runs with at least four genes for the LCA of all Rhizobiales species.

For each of the selected reconstructed gene runs, we scanned through all 22 leave-one-out reconstructions and determined if a similar enough gene run has also been produced, which is defined as sharing at least 80% of its genes with the original. If 18 or more leave-one-out reconstructions produced a similar enough gene run, we marked the original recovered, otherwise missed. During the analysis of the missed gene runs, we quickly realized that many gene runs are marked missed simply because they are broken into two or more fragments in the leave-one-out reconstructions by missing only a few gene pairs. We then loosened our criterion by marking a gene run recovered even if it has been broken into several fragments, as long as the longest two fragments contain together at least 80% of the genes in the original. The result is shown in Table 1.

**Table 1:** Leave-one-out stability test result. The table shows the difference in the number of gene runs as well as percentage under different cutoffs.

Number of fragments	1		2	
**gene pair cutoff**	0.85	0.9	0.85	0.9
**recovered**	77	103	107	118
**missed**	80	31	50	16
**total**	157	134	157	134
**percentage**	49.04%	76.87%	68.15%	88.06%

As we can see from the table, missing one genome has less impact on more conserved reconstruction. It also shows that when the gene-pair cutoff is set to 0.9, we recover 88% of the gene runs. This number should not be taken directly as an accuracy measure, since removing one genome from the data set will inevitably lead to lack of information to successfully reconstruct some of the original gene runs. It should be treated as a lower bound on the accuracy in the worst case. 

**Figure 3:**
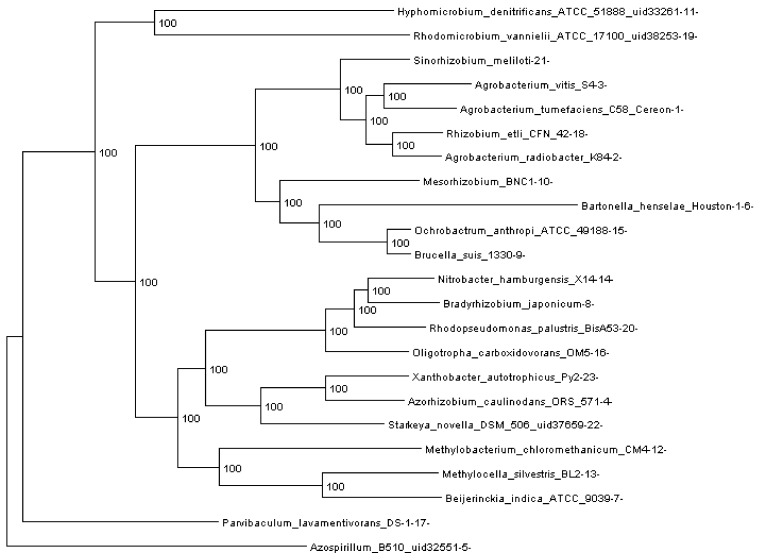
Rhizobiales phylogenomic species tree. The numbers shown at each branching are bootstrap score computed by RAxML [16] based on 100 runs. All numbers are 100, which indicates that this is a robust phylogenetic tree.

### 2.4. Phylogenomic Trees

In order to run REGEN on real genomes we created a phylogenomic tree for 23 Rhizobiales genomes (see Section 3.10) reconstructed with 109,273 genes (4,751 orthologous gene families, see next section). The resulting tree is shown in Figure 3. Each extant species (or input genome) was assigned an integer ID, which is the appended integer in the species name in the tree, so we can assign an easy and self-explanatory ID for each ancestor species. The ancestor ID reflects both child species. For example, ancestor 14_8_20 is the LCA of species 14, 8 and 20. A table of this assignment is provided in supplementary Table 1.

### 2.5. Orthologous Gene Identification and Refinement

For the Rhizobiales dataset OrthoMCL [17] identified 8,563 orthologous gene families, including 53,677 genes, which could be used directly for reconstruction. Gene families that were present in only one species were omitted. OrthoMCL also reported 3,125 mixed gene families (see Section 3.1), totaling 38,396 genes. These families underwent a refinement process using a method we developed [12]. At a conservative *p*-value of 0.01, an additional 3,892 orthologous gene families containing 18,606 genes were obtained. In total, 12,455 orthologous gene families with 72,283 genes were used as input for the reconstruction process.

The refinement of mixed gene families allows REGEN to partially compensate for the limitation it has in not dealing with gene duplications. Many genes that would otherwise be considered duplicates (paralogs) are separated in different families in the refinement process, thus making them available for reconstruction.

### 2.6. Running Time

The execution of REGEN on the Rhizobiales dataset took about three hours on a regular desktop computer. This time does not include the time to compute ortholog families.

### 2.7. Ancestral Gene Content Reconstruction

Gene content reconstruction was carried out by using the maximum likelihood reconstruction method implemented in BayesTraits [18]. To be consistent with the gene-pair reconstruction cutoff, all genes tagged with less than 0.9 probability were removed from further analysis. Details of the gene content reconstruction for each ancestor can be found in the supplementary material.

### 2.8. Ancestral Gene Run Reconstruction

The ancestral genome reconstructions were achieved through the use of gene runs and singleton genes. Gene runs provide information on the order of genes. Local synteny or conserved block information is useful information in genomics studies, because of correlation with operons. Table 3 lists the status of the reconstructed gene runs in the Rhizobiales data set. The last two columns show the absolute number of genes on gene runs and the respective percentage. The table shows that the quality of the reconstruction for the ancestor improves with the similarity of the genomes of child species. Higher similarity results in longer gene runs, which cover more genes, leaving fewer genes to be singleton genes in the genome. For example, the ancestral species 15_9 has its longest gene run with 121 genes and about 95% of its genes are in gene runs. On the other hand, the longest gene run in the ancestral species 13_7 only reaches 28 genes and about 33% of all its genes are singleton genes.

**Table 2:** Functional annotation of a particular reconstructed contiguous gene run in the LCA of the Rhizobiales group. Consensus column shows the number of genes that have been assigned with the corresponding annotation as well as the total number of genes in the family. Genes are sorted by the order on the chromosome.

Gene family ID	KEGG Entry	Function class	Definition	Consensus
1719	K02387	Cellular Processes; Cell Motility; Bacterial motility proteins, [BR:ko02035], Cellular Processes; Cell Motility; Flagellar assembly [PATH:ko02040]	flagellar basal-body rod protein FlgB	17/17
9901747	K02388	Cellular Processes; Cell Motility; Bacterial motility proteins, [BR:ko02035], Cellular Processes; Cell Motility; Flagellar assembly [PATH:ko02040]	flagellar basal-body rod protein FlgC	17/17
9901380	K02408	Cellular Processes; Cell Motility; Bacterial motility proteins, [BR:ko02035], Cellular Processes; Cell Motility; Flagellar assembly [PATH:ko02040]	flagellar hook-basal body complex protein FliE	17/17
9901964	K02392	Cellular Processes; Cell Motility; Bacterial motility proteins, [BR:ko02035] ,Cellular Processes; Cell Motility; Flagellar assembly [PATH:ko02040]	flagellar basal-body rod protein FlgG	17/17
1718	K02386	Cellular Processes; Cell Motility; Bacterial motility proteins, [BR:ko02035], Cellular Processes; Cell Motility; Flagellar assembly [PATH:ko02040]	flagella basal body P-ring formation protein FlgA	16/17
9903288	K02394	Cellular Processes; Cell Motility; Bacterial motility proteins, [BR:ko02035] ,Cellular Processes; Cell Motility; Flagellar assembly [PATH:ko02040]	flagellar P-ring protein precursor FlgI	16/17
1717	not annotated	N/A	N/A	N/A
9904536	K02393	Cellular Processes; Cell Motility; Bacterial motility proteins, [BR:ko02035], Cellular Processes; Cell Motility; Flagellar assembly [PATH:ko02040]	flagellar L-ring protein precursor FlgH	16/17
1828	K02415	Cellular Processes; Cell Motility; Bacterial motility proteins, [BR:ko02035]	flagellar FliL protein	16/16
9904106	K02419	Environmental Information Processing; Membrane Transport; Secretion system, [BR:ko02044],Cellular Processes; Cell Motility; Bacterial motility proteins, [BR:ko02035], Cellular Processes; Cell Motility; Flagellar assembly [PATH:ko02040]	flagellar biosynthetic protein FliP	17/17

**Table 3:** Contiguous gene run reconstruction overview of the Rhizobiales group. Length of the gene runs is measured in genes. The number-of-genes column shows the total number of genes on all gene runs and the percentage column shows the coverage of the gene runs.

Ancestor	number of gene runs	longest gene run length	number of genes	percentage
11_19_21_3_1_18_2_10_6_15_9_14_8_20_16_23_4_22_12_13_7_17	305	32	1321	79.87%
11_19_21_3_1_18_2_10_6_15_9_14_8_20_16_23_4_22_12_13_7	409	33	1716	85.16%
21_3_1_18_2_10_6_15_9_14_8_20_16_23_4_22_12_13_7	457	33	1894	85.43%
14_8_20_16_23_4_22_12_13_7	461	33	1869	82.70%
21_3_1_18_2_10_6_15_9	510	49	2394	84.95%
14_8_20_16_23_4_22	497	35	2019	85.23%
21_3_1_18_2	685	44	3688	88.31%
3_1_18_2	681	47	3918	90.03%
14_8_20_16	509	47	2775	91.28%
10_6_15_9	352	33	1561	77.62%
12_13_7	333	17	1024	56.83%
23_4_22	556	35	2336	83.01%
6_15_9	402	36	2153	95.94%
14_8_20	525	31	2513	74.55%
14_8	339	40	2638	91.95%
3_1	483	75	3863	92.70%
18_2	589	136	5280	94.57%
13_7	384	28	1272	67.55%
23_4	502	16	1933	76.28%
15_9	353	121	3615	94.63%
11_19	260	31	821	60.50%

### 2.9. Functional Annotation of Gene Runs

Functional annotation of one particular gene run for the reconstructed Rhizobiales LCA is given in Table 2 as an example; all other annotations can be found in the supplementary material, along with functional annotation for singleton genes.

### 2.10. Evolutionary History of Ancestral Gene Runs

**Figure 4:**
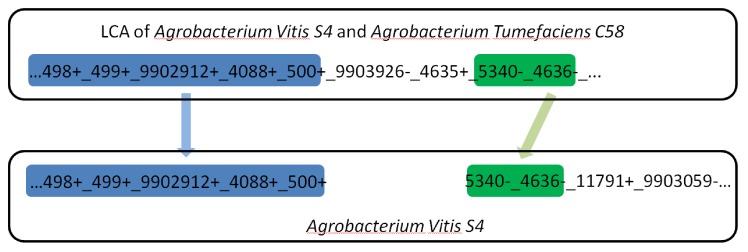
A long gene run on the main chromosome split into two smaller fragments during the evolutionary path from the LCA of *Agrobacterium vitis* S4 and *Agrobacterium tumefaciens* C58 to *Agrobacterium vitis* S4. Each number represents a gene and the underscore represents adjacency. +/- symbols represent the gene orientation determined during the reconstruction. Some genes on both ends are omitted for simplicity.

With the completion of the reconstruction of all ancestral gene runs, it is possible to formulate a hypothesis on what has happened to each gene run during a specific evolutionary path by analyzing the shared genes in the gene runs in both parent and child species. One example is shown in Figure 4.

All reconstructed scenarios for all evolutionary paths in the tree can be found in the supplementary material.

### 2.11. Replicon Reconstruction

Replicon reconstruction is the centerpiece of this study. We reconstructed the genome architecture of all ancestral species through analysis of the gene content of the child species and the outgroup. Based on the reconstructed replicons, replicon-scale evolutionary events can be predicted based on comparison of the genomes along each branch in the tree.

Only two ancestral genomes contain replicons qualified to be secondary chromosomes. A secondary replicon qualifies to be a chromosome in our model if it contains 5% or more core genes, with the fraction taken from the total number of core genes (for a definition of core gene see Section 3.1). The two ancestors are 15_9 (the ancestor of *Brucella suis* and *Ochrobactrum anthropi*), and 6_15_9 (the ancestor of 15_9 and *Bartonella henselae*). In the path from 10_6_15_9 to 6_15_9, a chromosomal split event divided the main chromosome into two chromosomes, and the new secondary chromosome was reconstructed as carrying a number of core genes. This property may have ensured the survival of this secondary chromosome in the extant species. It is also worth mentioning that the secondary chromosomes of *Agrobacterium radiobacter* K84 and *Agrobacterium vitis* S4 were not considered chromosomes in our model because they do not contain enough core genes.

**Figure 5:**
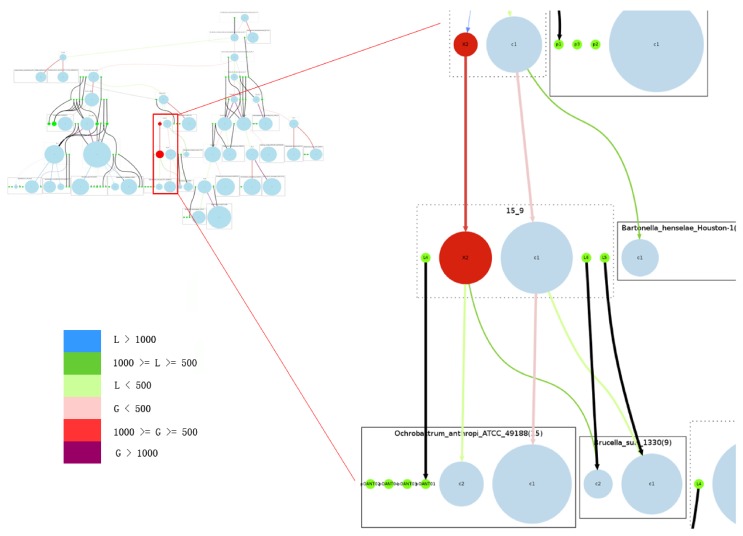
Overview of the complete reconstructed evolutionary history of the Rhizobiales group. Rectangles with solid edges represent input genomes and rectangles with dotted edges represent ancestral genomes. Replicons are represented as circles, with circle size proportional to replicon size (in number of genes) in the case of chromosomes; all plasmids are represented with same-sized circles. Chromosomes are shown in light blue, plasmids in green. The reconstructed secondary chromosomes are shown in red. Edge width corresponds to the strength of the inheritance relationships between replicons, and color shows the increase or decrease of chromosome sizes. Edges connected with plasmids are all marked black. On the right we provide a zoomed-in detail. Legend for color code: *L*: decrease in number of genes; *G*: increase in number of genes.

Figure 5 shows that in our reconstruction this group of Rhizobiales species constantly underwent plasmid split and plasmid merge, which could be true for most bacterial genomes due to the high frequency of recombination. A chromosome can easily pick up genes from a plasmid, and those genes could have come from a previous lateral gene transfer event. However, it is uncommon for a chromosome to undergo a replicon split and then lose some of its core genes.

### 2.12. Genome Architecture Evolution Reconstruction

The overview of the reconstruction of Rhizobiales species with the complete reconstruction process described above is summarized in Figure 5.

### 2.13. Comparison to Previous Work

We compared our results with those in Slater *et al*. [2] and Boussau *et al*. [1]. The former is more closely related to this study, because most of the input genomes are the same, and their goal was also to study replicon evolution.

Using a manual reconstruction, Slater *et al*. [2] identified a few conserved blocks that are shared by a group of Rhizobiales species. We mapped the species onto our tree and compared the identified conserved blocks in the reconstructed genome of the corresponding ancestor. After the mapping, the status of a conserved block identified by Slater *et al*. can be one of the following: (1) *identical*, meaning both blocks are identical; (2) *extended*, meaning the reconstructed gene run is longer; (3) *fragmented*, meaning the conserved block was mapped to more than one reconstructed gene run in our study; (4) *inconsistent*, meaning there is some difference between the conserved block and the reconstructed gene run in our study; and (5) *missing*, meaning it failed to be mapped to any reconstructed gene run. Out of the 31 conserved blocks in Slater *et al*. [2], eight are identical, 13 extended, four missing, five fragmented, and one inconsistent. All cases of discrepancy, including missing, fragmented, and inconsistent, are due to differences in the genome sets used in their work and ours. Details can be found in the supplementary material.

Among the results presented by Boussau *et al*. [1] are genome size increase and decrease (in terms of number of genes) along the phylogenetic tree for *α*-proteobacteria. Such results are directly comparable to our results, but only for the species analyzed in both studies. This comparison shows agreements in some cases and disagreements in others. For example, according to the scenario inferred by Boussau *et al*. [1], the genome of *Sinorhizobium meliloti* experienced a gain in genes between 500 and 1,000 from its LCA with *A. tumefaciens C58*, whereas in our study there was a decrease in the number of genes. The sizes of our reconstructed ancestral genomes are generally smaller when compared to those reconstructed by Boussau *et al*. [1], and we hypothesize this is due to the stringent probability cutoff we have used for NGP reconstruction.

The reconstructed LCA for Rhizobiales in this study contains several groups of essential genes. Overall, more than 500 genes are categorized as involved in metabolism in the KEGG Orthology [19]. There are 54 genes in the A-polymerase pathway (KO03010), and 24 genes in Aminoacyl-tRNA biosynthesis (KO00970). Details can be found in the supplementary material.

Boussau *et al*. [1] pointed out that their reconstructed ancestor (for all *α*-proteobacteria) has genes for glycolysis and a complete system for aerobic respiration system. A similar result is found in this study, in that the LCA Rhizobiales genome contains 22 genes in the Glycolysis/Gluconeogenesis pathway, covering 18 different KEGG Orthology function annotations.

One other prediction we can make on the ancestral phenotypic features is the mobility of the Rhizobiales LCA. The LCA was reconstructed as possessing 14 genes in the bacterial chemotaxis pathway and 47 genes in the flagella assembly pathway, which suggests that it was capable of moving around and sensing the chemical signals in the surrounding environment. Details can be found in the supplementary material.

## 3. Experimental Section

### 3.1. Definitions

The inputs for REGEN are a set of annotated bacterial genomes of organisms that are closely related (typically in the same bacterial order or lower down in the taxonomy). Homology between genes is determined by sequence similarity. An *homologous gene family* is a gene family that contains at least two genes. An *orthologous gene family* is an homologous gene family that contains at most one gene from each species. A *mixed gene family* is an homologous gene family that contains more than one gene from the same species. A *singleton gene* is a gene that does not have an homologous counterpart in any other genome. A *core gene* is a gene that occurs in all genomes in the set. Each gene family is assigned a unique ID. The *gene family alphabet* Σ is the set of all these IDs plus *, which is used to represent a singleton gene. A *gene run* is a finite string over Σ from a genome that does not include *. A *genome* is a set of strings over Σ. A *replicon* in a genome is one of the strings of a genome. Every genome has a *main chromosome* that is the largest replicon. Some genomes have a *secondary chromosome*, *i.e*., a large replicon containing more than a certain threshold of core genes (5 % of the total number of core genes). A *phylogenomic tree* is a tree built based on the concatenation of separate multiple alignments of protein sequences (encoded by genes in a genome appearing exactly once on all input genomes). The *last common ancestor* of a set of extant species is the most recent shared ancestor of those species.

### 3.2. Species Tree Reconstruction

As mentioned, REGEN needs a reliable species tree as input. In cases where such a tree is not available, a phylogenomic tree based on the supermatrix approach [20] can be built, as briefly described next. An all-against-all BLAST search [21] between all protein sequences in the annotated input genomes is performed. The BLAST output is then fed to OrthoMCL [17] with default values to identify orthologous gene families. Families with only one member in all the species are selected for the tree reconstruction. MUSCLE [22] is used to create multiple sequence alignment for each family, and Gblocks [23] with parameter setting -b5 = h is used for alignment trimming. Trimmed alignments are concatenated and fed to RAxML [16] with default values for final species tree reconstruction.

### 3.3. Homologous Gene Family Identification and Refinement

Before REGEN can be run, orthologous gene families must be identified in the input genomes. It is a requirement that no family may contain paralogous genes. Because homologous family identification programs such as OrthoMCL [17] and MULTIPARANOID [24] produce homologous gene families with paralogous genes, we run a refinement process [12] on the output of OrthoMCL to discard paralogous genes and at the same time identify more orthologs. The result of this process is that each orthologous gene receives an identifier corresponding to the family to which it belongs. Each replicon can then be represented by an ordered array (or string) of such identifiers. Singleton genes are represented by asterisks.

### 3.4. Ancestral Genome Reconstruction

REGEN can do gene content reconstruction by two different methods: maximum likelihood (ML) and maximum parsimony (MP). For ML we used the program BayesTraits [18]. Our MP method is a slightly modified version of the method described in [5].

In our implementation, each gene is represented by two symbols, one for each end (5′ and 3′). This notation allows us to encode both the adjacency and orientation information for each gene. This two-node notation also reduces the complexity of the gene run reconstruction algorithm described next. Each adjacent gene pair is treated as a feature for a genome, and the status of such features on the ancestral genomes is reconstructed using the same method as described in the gene content reconstruction.

After the successful reconstruction of all the NGPs, the following algorithm is designed to reconstruct gene runs for each ancestral genome. The algorithm starts from a random pair, iteratively identifies all other pairs that may be connected, and builds an undirected connected graph with all these pairs. Each edge is weighted as the reciprocal of the probability of having the particular NGP in the ML-based reconstruction and 1 in the MP-based reconstruction. All edges connecting both ends of a single gene have the weight set to 1 in both methods. Then the algorithm identifies a minimum spanning tree (MST) in this graph using Kruskal’s algorithm [25] to obtain a subgraph without cycles. The Bellman-Ford algorithm [26] is then run on the MST to calculate scores for paths between all node pairs. A legitimate path with the lowest score is then identified and recorded as a reconstructed gene run. A path is legitimate if and only if inter-gene edges and intra-gene edges interleave. All nodes included in the path are removed following the identification and the original MST is reduced and may split into two or more fragments. The Bellman-Ford algorithm is run on each fragment and the process is repeated until all nodes are removed or a new fragment consists of only one gene.

The establishment of such relationships is based on the following graph-based algorithm, designed to utilize the concept of a *group*, defined as a collection of genes that share the same inheritance pattern. Genes are considered co-inherited if they reside together on a single replicon in both genomes. For example, if genes *a*, *b*, and *c* are on one replicon in two species *X* and *Y*, then they are considered to be in the same group. The reconstruction assumes that co-inherited genes are more likely to be on the same replicon in the ancestral genome, because the probability of having multiple genes moving to the same replicon due to independent evolutionary events is low. The idea behind the algorithm is first to divide the genes on the replicons in the extant species into co-inherited groups and then determine which groups are likely to be on the same replicon in the ancestral species; and then finally merge the groups back into replicons according to the linkages established during the reconstruction process. Essentially, only genes that share some inheritance pattern in both the out-group and in-group species are merged into a replicon, which turns out to be a quite stringent criterion.

**Figure 6:**
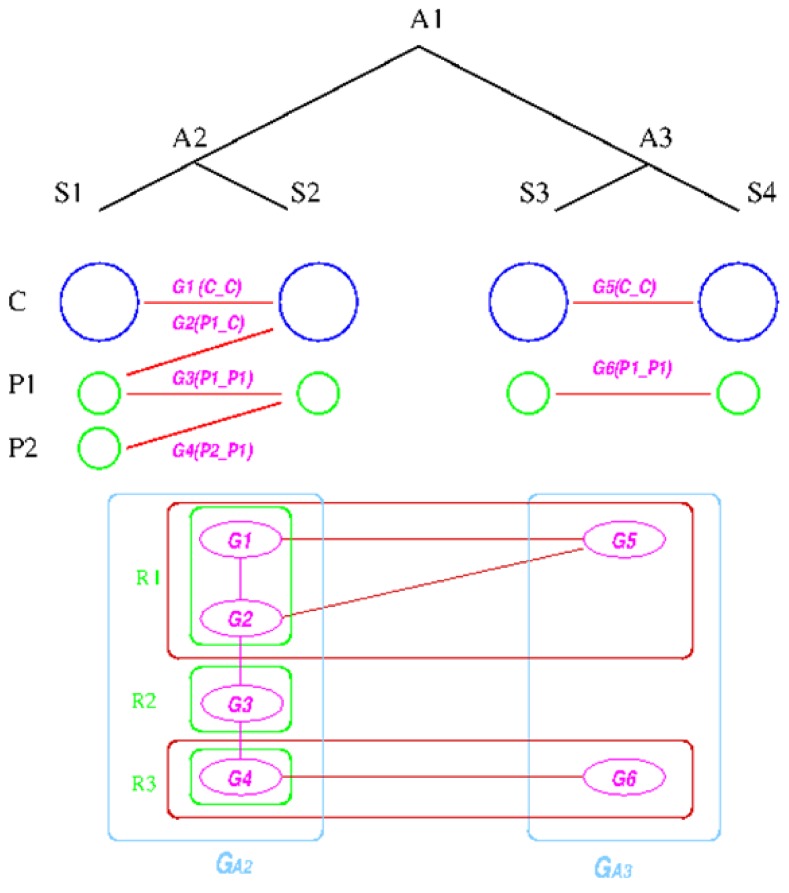
Replicon architecture reconstruction example. Blue circles represent main chromosomes, green circles plasmids, and purple ovals gene groups. Red boxes represent identified connected components in the group graph and green box final replicon architecture reconstruction result.

We explain the algorithm with the following example, in which the four extant species are named 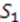, 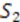, 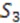, and 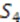 and ancestral species are named 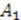, 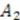 and 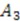 respectively (see Figure 6). For simplicity, all main chromosomes in the four extant species are named *C* and the plasmids are named 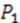 and 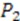. Here, we will decide the genome architecture of 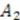, using 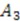 as an outgroup. Notice that the genome architecture of 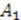 cannot be determined without adding more species as outgroups.

The algorithm starts by computing co-inherited gene groups for each ancestral species in the tree in a bottom-up fashion. In our example, four groups are identified for 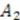 and two for 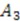. Group graph 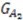 and 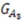 are created for the two ancestral species, with each group as a vertex. Edges are added if two groups shared a replicon in their co-inheritance pattern, such as 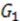 (C_C) and G_2_ (P1_C) (shared C in 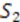). Then, the relationship between groups in 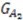 and 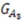 is computed, and edges are added if the number of shared genes exceeds a certain cutoff, denoted by dark red edges. For each connected component in the outgroup group graph, which is 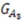 in the example, we identify all vertices in the target species group graph. For these identified vertices, we will merge the ones that are connected back into replicons. Any unmerged group will form its own replicon, such as 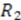. Final genome architecture for 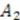 is shown by green ovals.

After replicon reconstruction, all genes are tagged with their own replicon information.

### 3.5. Reconstructed Replicon Merge

During the application of the above algorithm to both real and simulated data, we noticed that it tends to produce more replicons than there really are. We also noticed that many long reconstructed gene runs contain genes that have been assigned to different replicons. Based on these observations, we designed an extra step that merges replicons based on the discrepancy of replicon information for genes in the gene runs.

The algorithm starts by selecting a set of reconstructed gene runs with a length limit, which is set to 4 genes for the data shown in this work. Then it checks for discrepancies of the gene location information in this set of gene runs. Discrepancy is defined as genes on the same gene run that are assigned to different replicons. From each reconstructed gene run in this selected collection, we evaluate the relative signal strength of replicon merging, which is defined as

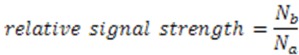

where 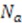 is the number of genes assigned to the most frequently occurring replicon and 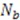 the number of genes assigned to another replicon.

Gene runs with extremely low signals are ignored. We then assign the length of the gene run as the strength of the merging proposal supported by this specific gene run. The strength of all gene runs for merging the same pair of replicons are summed and the result is defined as the absolute signal strength of the merging proposal at the species level. All merging proposals are gathered together for all ancestral species, and a *K*-means clustering is performed on both absolute and relative signal strength, with 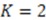. The values that divide the result clusters are chosen as the line between accepting or rejecting merging proposals. The algorithm proceeds in a bottom-up fashion and ends when all merging proposals are either accepted or rejected.

### 3.6. Chromosome Restoration

With the replicons for each ancestral genome being reconstructed, it is time to distinguish chromosomes from plasmids. The notion of chromid [27] is not considered here. This process is carried out using core genes. The main chromosome is assigned to the replicon with the most core genes. For secondary chromosome assignment, a minimum number of core genes (5 % of the total number of core genes by default) have to reside on the replicon.

### 3.7. Ancestral Evolutionary Event Reconstruction

By comparing the gene runs and gene content between parent and child species, we can infer a large number of different evolutionary events on both the gene and replicon scales, such as gene loss, gene gain, replicon merge, and replicon loss. We can even infer gene reversal events, if they happened within a reconstructed gene run.

### 3.8. Ancestral Gene Run and Genome Function Annotation

The Kyoto Encyclopedia of Genes and Genomes (KEGG) [19] was used as the source of function annotation. To determine the potential phenotypic features of an ancestral species, we need to first determine the function of as many of its genes as possible. To achieve this, we assign the most frequently occurred functional annotation among all family members to the function annotation for the orthologous gene family. The determined function is later transferred to the gene in the ancestral genome. After the completion of this annotation process for as many genes in the ancestral genome as possible, we determine possible ancestral phenotypic features by examining the gene content with their function annotation of an ancestral genome.

Due to the close resemblance between reconstructed consecutive gene runs and operons in bacterial genomes, we not only used the annotated genes to infer the functional roles played by some gene runs but also validated these reconstructed gene runs by checking the consistency among the members they contain.

### 3.9. Simulated Datasets

We have evaluated REGEN in part using simulated data generated by our own simulator [13]. The simulator generates a bifurcating tree starting with a user-provided start genome parameter values. Along the tree the following events can occur: gene gain and loss, reversals, gene duplication, gene translocation, and gene transposition. At the replicon level the following events are possible: gain, merge, split, and loss. In [13] we show that the simulator generates simulated data sets with properties similar to those found in real data sets.

The gene pair occurrence likelihood cutoff that determines what gene pairs are present in an ancestral genome is an important parameter to set in the ML based method. On the other hand, the gene occurrence likelihood cutoff that determines what genes are present in an ancestral genome has minimum impact on the results, since we are more focused on reconstructed gene runs that are reconstructed from gene pairs exclusively. With this simulator, we were able to compare results with different settings and make an informed choice on the parameter settings for the real data set. 

### 3.10. Genomes

The group of Rhizobiales species was chosen because of their complex genome architecture, including secondary chromosomes and several large plasmids. The 22 species from the Rhizobiales order include *Agrobacterium tumefaciens* C58, *Agrobacterium vitis* S4, *Agrobacterium radiobacter* K84, *Azorhizobium caulinodans* ORS 571, *Bartonella henselae* Houston-1, *Beijerinckia indica* ATCC 9039, *Bradyrhizobium japonicum*, *Brucella suis* 1330, *Mesorhizobium* BNC1, *Hyphomicrobium denitrificans* ATCC 51888, *Methylobacterium chloromethanicum* CM4, *Methylocella silvestris* BL2, *Nitrobacter hamburgensis* X14, *Ochrobactrum anthropi* ATCC 49188, *Oligotropha carboxidovorans* OM5, *Parvibaculum lavamentivorans* DS-1, *Rhizobium etli* CFN 42, *Rhodomicrobium vannielii* ATCC 17100, *Rhodopseudomonas palustris* BisA53, *Sinorhizobium meliloti*, *Starkeya novella* DSM 506, and *Xanthobacter autotrophicus* Py2. *Azospirillum* B510 was chosen as an outgroup. The choice of the outgroup species was made based on the phylogenetic tree presented in [28]. All genome sequences were downloaded from the NCBI Genbank FTP site. 

## 4. Conclusions

We have presented the first automated method for ancestral genome reconstruction at both the gene and replicon levels without prior assumptions on the ancestral genome replicon architecture. It is also the first method that can reconstruct gene runs for ancestral genomes with fully resolved strand information in bacteria with functional annotation using external databases. We have also modified and improved the original NGP-based method so it does not require a reference genome, correctly handles strand information, and employs a two-step occurrence uncertainty resolution process. Based on the reconstructed genomes, REGEN can also propose possible scenarios on the evolutionary events for both gene runs and replicons along the branches in the species tree.

It is important to mention some of the assumptions and simplifications we have made in designing REGEN. First, the replicon reconstruction algorithm assumes that, in the two child species, groups sharing more genes are more likely to be on the same replicon in the ancestral genome than in separate replicons. This could be unrealistic if some large scale evolutionary events affected a large number of genes in an uneven fashion. Second, the system will only work with bifurcating trees. Third, we do not have a concept of time. Due to the lack of data to determine mutation rates of events at all different scales, including gene-, replicon-, and genome-scale, we decided to leave the concept of time out of the scope of the current study. Without it, we cannot determine which ancestral species co-existed at the same point of time, which means that we cannot reconstruct evolutionary events that involved more than one ancestral species, such as horizontal gene transfer. On the other hand, these very simplifications were necessary to make this method feasible and capable of handling dozens of genomes in a reasonable time.

## Supplementary Files

Supplementary File 1 PDF-Document (PDF, 185 KB)
