# Supplementary material for: REGEN: Ancestral Genome Reconstruction for Bacteria
Source: Genes (Basel). 2012 Jul 18;3(3):423–43. doi: 10.3390/genes3030423 (PMC3899994; doi:10.3390/genes3030423)

Supplemental material

Kuan Yang, Lenwood S. Heath, João C. Setubal

March 29, 2012

1 supplementary tables

Table 1: Integer ID for all Rhizobiales species

Species Name	Integer ID
Agrobacterium_tumefaciens_C58_Cereon	1
Agrobacterium_radiobacter_K84	2
Agrobacterium_vitis_S4	3
Azorhizobium_caulinodans_ORS_571	4
Azospirillum_B510_uid32551	5
Bartonella_henselae_Houston-1	6
Beijerinckia_indica_ATCC_9039	7
Bradyrhizobium_japonicum	8
Brucella_suis_1330	9
Mesorhizobium_BNC1	10
Hyphomicrobium_denitrificans_ATCC_51888_uid33261	11
Methylobacterium_chloromethanicum_CM4	12
Methylocella_silvestris_BL2	13
Nitrobacter_hamburgensis_X14	14
Ochrobactrum_anthropi_ATCC_49188	15
Oligotropha_carboxidovorans_OM5	16
Parvibaculum_lavamentivorans_DS-1	17
Rhizobium_etli_CFN_42	18
Rhodomicrobium_vannielii_ATCC_17100_uid38253	19
Rhodopseudomonas_palustris_BisA53	20

Sinorhizobium_meliloti	21
Starkeya_novella_DSM_506_uid37659	22
Xanthobacter_autotrophicus_Py2	23

Table 2: Genome architecture for the Rhizobiales group

Species	# of chromosomes	# of plasmids
Sinorhizobium_meliloti	1	2
Azospirillum_B510_uid32551	1	6
Rhodopseudomonas_palustris_BisA53	1	0
Beijerinckia_indica_ATCC_9039	1	2
Azorhizobium_caulinodans_ORS_571	1	0
Oligotropha_carboxidovorans_OM5	1	0
Parvibaculum_lavamentivorans_DS-1	1	0
Bartonella_henselae_Houston-1	1	0
Xanthobacter_autotrophicus_Py2	1	1
Methylocella_silvestris_BL2	1	0
Rhizobium_etli_CFN_42	1	6
Bradyrhizobium_japonicum	1	0
Ochrobactrum_anthropi_ATCC_49188	2	4
Starkeya_novella_DSM_506_uid37659	1	0
Methylobacterium_chloromethanicum_CM4	1	2
Nitrobacter_hamburgensis_X14	1	3
Agrobacterium_tumefaciens_C58_Cereon	2	2
Brucella_suis_1330	2	0
Hyphomicrobium_denitrificans_ATCC_51888_uid33261	1	0
Rhodomicrobium_vannielii_ATCC_17100_uid38253	1	0
Agrobacterium_radiobacter_K84	2	3
Mesorhizobium_BNC1	1	3
Agrobacterium_vitis_S4	2	5

Table 3: Gene content reconstruction

Ancestor ID	Gene on chromosomes	Genes on plasmids	total
11_19_21_3_1_18_2_10_6_15_9_14 _8_20_16_23_4_22_12_13_7_17	1435	219	1654
11_19_21_3_1_18_2_10_6_15_9_14 _8_20_16_23_4_22_12_13_7	1446	569	2015
21_3_1_18_2_10_6_15_9_14__20 _16_23_4_22_12_13_7	1457	760	2217
14_8_20_16_23_4_22_12_13_7	1272	988	2260
21_3_1_18_2_10_6_15_9	1955	863	2818
14_8_20_16_23_4_22	1287	1082	2369
21_3_1_18_2	2549	1627	4176
3_1_18_2	2464	1888	4352
14_8_20_16	2560	480	3040
10_6_15_9	1754	257	2011
12_13_7	1245	557	1802
23_4_22	2603	211	2814
6_15_9	2146	98	2244
14_8_20	2940	431	3371
14_8	2479	390	2869
3_1	3507	660	4167
18_2	4941	642	5583
13_7	1636	247	1883
23_4	2271	263	2534
15_9	3358	462	3820
11_19	1221	136	1357

Table 4: The distribution of core genes in the Rhizobiales data set

Sinorhizobium_meliloti		
	c1	584
	pSymA	0
	pSymB	3

Azospirillum_B510_uid32551		
	c1	527
	pAB510a	18
	pAB510b	0
	pAB510c	16
	pAB510d	17
	pAB510e	9
	pAB510f	0
Rhodopseudomonas_palustris_BisA53		
	c1	587
Beijerinckia_indica_ATCC_9039		
	c1	587
	pBIND01	0
	pBIND02	0
Azorhizobium_caulinodans_ORS_571		
	c1	587
Oligotropha_carboxidovorans_OM5		
	c1	587
Parvibaculum_lavamentivorans_DS-1		
	c1	587
Bartonella_henselae_Houston-1		
	c1	587
Xanthobacter_autotrophicus_Py2		
	pXAUT01	0
	c1	587
Methylocella_silvestris_BL2		
	c1	587
Rhizobium_etli_CFN_42		
	c1	585
	p42a	0
	p42b	0
	p42c	0
	p42d	1
	p42e	0
	p42f	1
Bradyrhizobium_japonicum		

	c1	587
Ochrobactrum_anthropi_ATCC_49188		
	c1	549
	c2	38
	pOANT01	0
	pOANT02	0
	pOANT03	0
	pOANT04	0
Starkeya_novella_DSM_506_uid37659		
	c1	587
Methylobacterium_chloromethanicum_CM4		
	c1	587
	pMCHL01	0
	pMCHL02	0
Nitrobacter_hamburgensis_X14		
	c1	587
	p1	0
	p2	0
	p3	0
Agrobacterium_tumefaciens_C58_Cereon		
	c1	523
	c2	64
	At	0
	Ti	0
Brucella_suis_1330		
	c1	533
	c2	54
Hyphomicrobium_denitrificans_ATCC_51888_uid33261		
	c1	587
Rhodomicrobium_vannielii_ATCC_17100_uid38253		
	c1	587
Agrobacterium_radiobacter_K84		
	c1	587
	c2	0
	pAgK84	0
	pAtK84b	0

	pAtK84c	0
Mesorhizobium_BNC1		
	c1	586
	p1	1
	p2	0
	p3	0
Agrobacterium_vitis_S4		
	c1	580
	c2	7
	pAtS4a	0
	pAtS4e	0
	pAtS4c	0
	pTiS4	0
	pAtS4b	0

Table 5: The distribution of the core genes in all ancestral genomes and secondary chromosome assignment

**ancestor**	**replicon**	**Number of CG**
6_15_9		
	c1	524
	c2	51
	U	0
10_6_15_9		
	c1	579
	L2	0
	U	0
21_3_1_18_2		
	c1	575
	L3	0
	L5	0
	L6	0
	L7	0

Continued on next page

Table 5 – continued from previous page

ancestor	replicon	Number of CG
	L9	0
	L10	0
	L11	0
	L12	3
	L14	0
	L16	0
	L18	0
	U	0
21_3_1_18_2_10_6_15_9		
	c1	574
	L3	0
	L4	0
	L5	0
	L7	0
	L8	0
	L10	0
	U	2
14_8_20_16_23_4_22_12_13_7		
	c1	546
	L5	0
	L6	0
	U	0
14_8_20_16		
	c1	577
	L3	0
	L4	0
	L5	0
	U	0
13_7		
	c1	577
	U	0
23_4		
	c1	585

Continued on next page

Table 5 – continued from previous page

ancestor	replicon	Number of CG
	L4	0
15_9		
	c1	532
	c2	55
	L4	0
	L5	0
	L6	0
	U	0
11_19		
	c1	420
12_13_7		
	c1	501
	R2	0
	L4	0
	U	0
3_1		
	c1	580
	R1	0
	L6	1
	U	0
3_1_18_2		
	c1	580
	R1	0
	R2	0
	R3	0
	R4	0
	L8	0
	L9	0
	U	0
	L7	0
23_4_22		
	c1	558
	U	0

Continued on next page

Table 5 – continued from previous page

ancestor	replicon	Number of CG
14_8_20		
	c1	560
	R2	0
	L3	0
	U	0
14_8		
	c1	584
	L2	0
	U	0
21_3_1_18_2_10_6_15_9_14_8_20_16_23_4_22_12_13_7		
	c1	557
	R1	0
	L5	0
	L7	0
	U	0
18_2		
	c1	584
	R4	0
	L6	0
	L7	0
	U	0
14_8_20_16_23_4_22		
	c1	542
	R1	0
	R2	4
	R3	1
	L5	0
	L6	0
	U	0
11_19_21_3_1_18_2_10_6_15_9_14_8_20_16_23_4_22_12_13_7		
	c1	551
	R4	4
	U	0

Continued on next page

Figure 1: Genome coverage achieved by reconstructions at different gene pair cutoff.

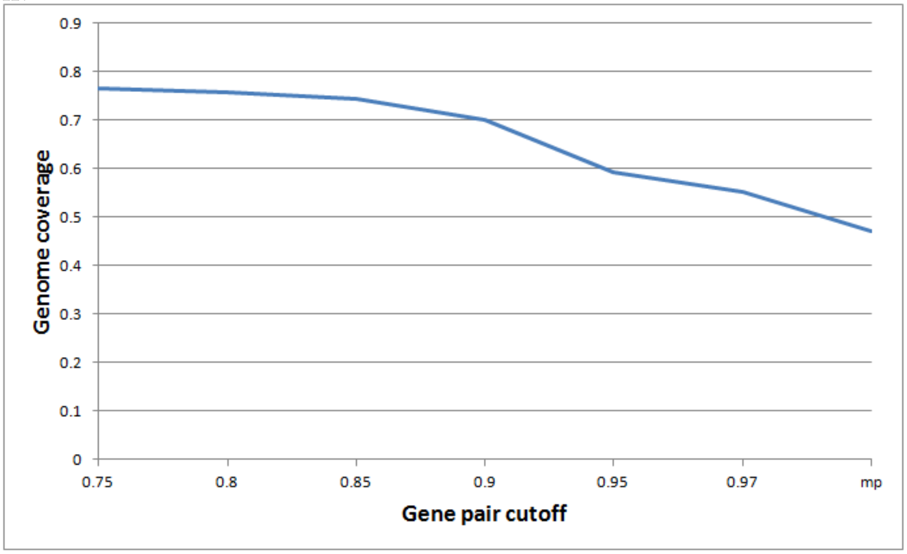

Table 5 – continued from previous page

ancestor	replicon	Number of CG
11_19_21_3_1_18_2_10_6_15_9_14_8_20_16_23_4_22_12_13_7_17		
	c1	545
	U	0

2 Supplemental Figures

Genome Coverage

Genome coverage is calculated with simulated data. By comparing the reconstructed gene runs of the LCA with the true genome, we are able to calculate how much of the genome is covered by the reconstructed gene runs. The result is shown in the following figure.

Setting the gene pair occurrence cutoff to a lower value naturally results in more gene pairs which then cover more of the genome. It is worth noticing that the coverage decrease is not observed until the setting reach 0.95 and

Figure 2: Longest gene run length and correct longest gene run length in the reconstructions at different cutoff.

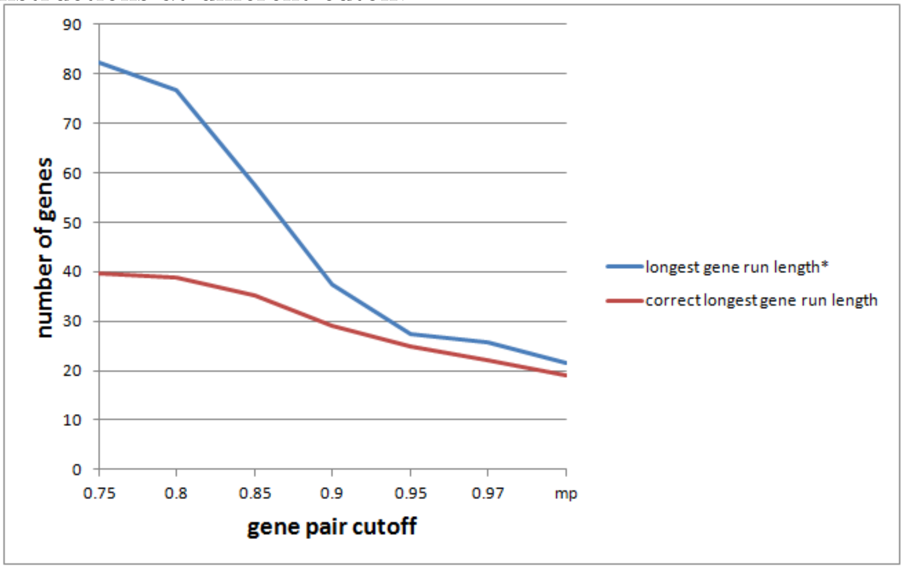

MP achieves the least genome coverage. The result is shown in Figure 1.

Longest reconstructed gene run length

The longest gene run is of particular interest because of the information they can provide. Figure 2 show the length of the longest reconstructed gene run at different settings. The length only counts the corrected mapped part when discrepancy occurs.

Conserved Blocks Reconstruction

One of things that attract a lot of our attention is the conserved blocks. Conserved blocks are contiguous runs of genes on the genome that carry important functions and thus more conserved than other parts of the genome. We are extremely interested to see how much of the conserved blocks can be restored by our reconstruction. Figure 3 shows the comparison of the percentages of conserved blocks that have been completely reconstructed or missed in different reconstructions.

0% means complete absent in the reconstruction. <20% means the percentage of the conserved blocks that have been reconstructed less than 20%. <40% means the percentage of the conserved blocks that have been reconstructed between 20% and 40%, and so on and so forth. 100% means the

Figure 3: Conserved blocks reconstruction status.

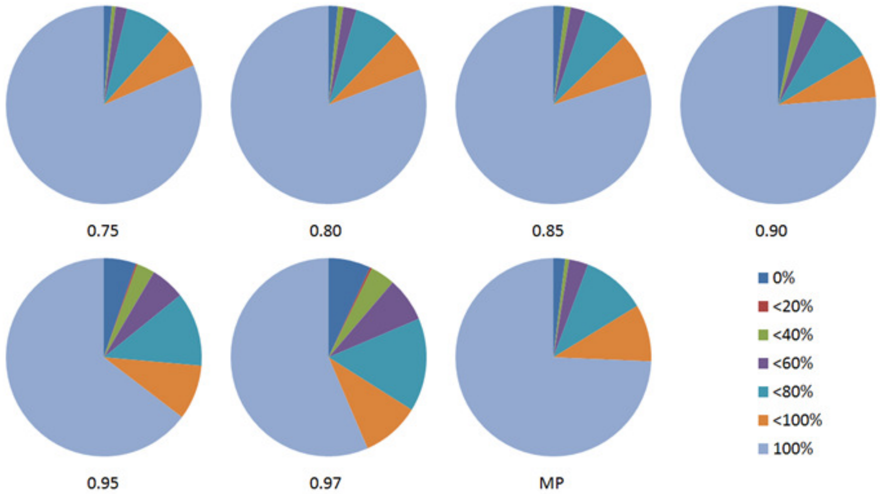

percentage of conserved blocks that have been completely reconstructed.

Gene Pair precision V.S. recall measure

Based on simulated data, we are able to compare map the reconstructed gene pairs for each ancestral genome to the actual genomes and calculate precision and recall, which are then plotted in Figure 4.

Replicon reconstruction accuracy

As the first ancestral genome reconstruction system with the ability to target at replicon-scale, the accuracy of such reconstructions is of extremely interest. With the simulated data, we are able to accurate measure the performance of the system with the following metrics. For an ancestral genome, we defined a replicon matched if there is a reconstructed replicon that shares a considerate amount of genes with it, otherwise missed. For a reconstructed ancestral genome, we defined a replicon extra if it cannot be mapped to any replicon in the corresponding ancestral genome or partial if it is mapped to an already matched replicon. To be conservative, if a reconstructed replicon shared a considerate amount of genes with more than one replicon in the actual genome, we only retain the strongest link and the only replicons are marked missed. The four measures are plotted in the following figure. Gene pair cutoff and gene cutoff were set to 0.9 with the consideration of all the information retrieved from simulation tests above.

Figure 4: Precision and recall for different reconstructions.

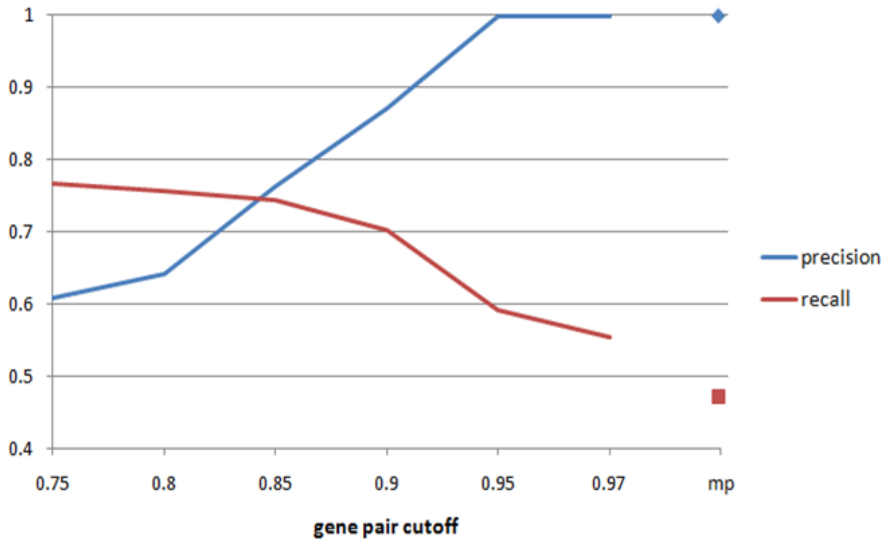

Figure 5: Replicon reconstruction accuracy.

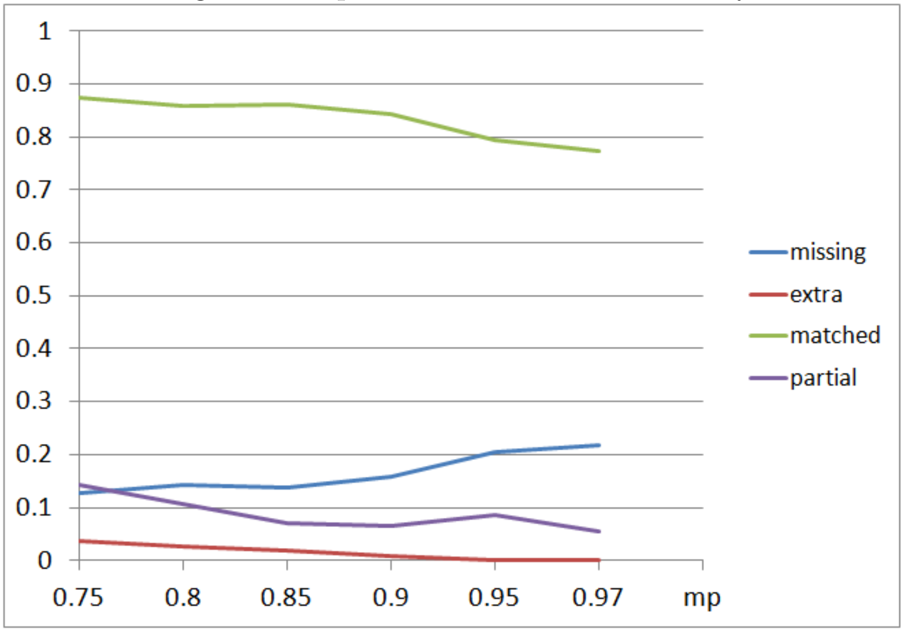

Supplement: Supplementary File 1 — PDF-Document (PDF, 185 KB) [file genes-03-00423-s001.pdf]
